# Western Blotting Inaccuracies with Unverified Antibodies: Need for a Western Blotting Minimal Reporting Standard (WBMRS)

**DOI:** 10.1371/journal.pone.0135392

**Published:** 2015-08-19

**Authors:** Jennifer E. Gilda, Rajeshwary Ghosh, Jenice X. Cheah, Toni M. West, Sue C. Bodine, Aldrin V. Gomes

**Affiliations:** 1 Department of Neurobiology, Physiology, and Behavior, University of California Davis, Davis, CA, United States of America; 2 Department of Pharmacology, University of California Davis, Davis, CA, United States of America; 3 Department of Physiology and Membrane Biology, University of California Davis, Davis, CA, United States of America; Florida International University Bimolecular Sciences Institute, UNITED STATES

## Abstract

Western blotting is a commonly used technique in biological research. A major problem with Western blotting is not the method itself, but the use of poor quality antibodies as well as the use of different experimental conditions that affect the linearity and sensitivity of the Western blot. Investigation of some conditions that are commonly used and often modified in Western blotting, as well as some commercial antibodies, showed that published articles often fail to report critical parameters needed to reproduce the results. These parameters include the amount of protein loaded, the blocking solution and conditions used, the amount of primary and secondary antibodies used, the antibody incubation solutions, the detection method and the quantification method utilized. In the present study, comparison of ubiquitinated proteins in rat heart and liver samples showed different results depending on the antibody utilized. Validation of five commercial ubiquitin antibodies using purified ubiquitinated proteins, ubiquitin chains and free ubiquitin showed that these antibodies differ in their ability to detect free ubiquitin or ubiquitinated proteins. Investigating proteins modified with interferon-stimulated gene 15 (ISG15) in young and old rat hearts using six commercially available antibodies showed that most antibodies gave different semi-quantitative results, suggesting large variability among antibodies. Evidence showing the importance of the Western blot buffer and the concentration of antibody used is presented. Hence there is a critical need for comprehensive reporting of experimental conditions to improve the accuracy and reproducibility of Western blot analysis. A Western blotting minimal reporting standard (WBMRS) is suggested to improve the reproducibility of Western blot analysis.

## Introduction

Western blotting is a technique that was developed in 1979 [1] and is now a commonly used technique in biomedical research. This method offers many advantages over other techniques for detecting and semi-quantifying target proteins, allowing the detection of a single target out of thousands of proteins as well as obtaining molecular weight information about the target protein in the same experiment [2]. The main disadvantage of Western blotting is that this technique requires a specific antibody to a target protein; thus many protein targets cannot be investigated because of the lack of specific antibodies. However, the number of antibodies available for Western blotting is expanding at a rapid pace as the production costs have decreased. A search of the internet on August 1^st^, 2014 showed that > 50,000 monoclonal and > 160,000 polyclonal antibodies are available from three companies for which the total number of antibodies available were listed on their websites (Santa Cruz Biotechnology, Aviva Systems Biotechnology and Abnova). The largest antibody search engine, CiteAb (www.citeab.com) has over 2.1 million antibodies listed as of April 2015. According to the Antibody Resource website (http://www.antibodyresource.com/onlinecomp.html), there are at least 200 companies that sell antibodies (April, 2015). The labome website (http://www.labome.com/method/Antibody-Companies.html) lists at least 316 companies that sell antibodies (April 2015). A major demand for new antibodies comes from the field of proteomics, where Western blot analysis is often used to validate differentially regulated proteins. However, the lack of highly specific antibodies is a common problem [3–9].

An investigation using one of the most commonly utilized commercial antibodies for the cannabinoid CB2 receptor showed that the common practice of only validating antibodies with positive controls is insufficient to ensure antibody reliability [10] (S1 Table). Evaluation of nine commercially available anti-CCR5 (CD195) monoclonal antibodies showed that three antibodies displayed substantial background binding to CCR5 negative cells [11]. In an important study that investigated more than 200 antibodies against 57 different histone modifications in *Drosophila melanogaster*, *Caenorhabditis elegans* and human cells, more than 25% of the antibodies failed Western blot or dot blot specificity tests [12]. These investigations all show that more rigorous testing of antibodies is needed.

Western blotting itself has also gotten less expensive, allowing more labs to utilize this technique. As more labs use Western blotting, more antibodies are being purchased. While it is now fairly straightforward to produce antibodies, determining the usefulness of each antibody requires a significant investment of time and money, as the number of applications of an antibody is numerous. As such the research community has experienced substantial problems with obtaining accurate results since most antibodies are made available for sale with limited validation. In many cases researchers must use an antibody with no reliable information regarding its usefulness for their application. In these cases the researcher is forced to validate commercially available antibodies for their application or generate their own antibody. Unfortunately, in our experience > 50% of commercially available antibodies are of poor quality and should not be utilized for semi-quantitative Western blot analysis. A survey done by 1DegreeBio of 400 antibody users reported that 48% of researchers found that at least half of the antibodies they purchased did not work as expected (http://blog.scienceexchange.com/2012/03/guest-post-alexandra-hodgson/). Another study done in 2008 showed that <50% of around 6,000 routinely used commercial antibodies recognized only their specified targets [13]. Although some of the error is likely due to experimental error, a substantial proportion is due to poor quality antibodies. Over the last few years several websites have been developed so the user community can provide feedback about antibody quality. These websites include the antibody validation database (http://compbio.med.harvard.edu/antibodies/), antibody advisor (http://www.antibody-adviser.org/pages/home), antibodypedia (http://www.antibodypedia.com/), pABmABs (http://pabmabs.com/wordpress/), and 1degreebio (http://1degreebio.org/). Unfortunately, these websites have not been as helpful as expected because relatively few researchers provide feedback, and the feedback provided is limited regarding the experimental conditions.

One way to improve the reproducibility of Western blot analysis is to improve the quality of the antibodies. Antibodies produced in animals sometimes give questionable results. Small scale experiments have suggested that recombinantly produced antibodies would improve reproducibility [14]. Pilot programs initiated in 2010 by the US National Institutes of Health (NIH), the Protein Capture Reagents Program, and by the European Union (EU), called Affinomics, have made attempts to scale up recombinant technologies, including recombinant antibodies that would help improve reproducibility [15]. However, this will take considerable time, and even when recombinant antibodies are available for most targets, other factors that affect the reproducibility of Western blot analysis still need to be taken into account. These factors include loading too much protein on polyacrylamide gels, using un-optimized buffers or blocking reagents, and lack of proper controls. Therefore, a major problem with Western blotting is not the method itself but the use of sub-standard antibodies and sub-standard techniques. To determine all the problems that exist and the best solutions, detailed information in publications are needed. The lack of Western blotting information present in journal articles currently prevents researchers from reproducing original results and does not prevent poor quality antibodies from being used by other laboratories. In some published papers the lack of reporting standards is apparent, as evident by the failure of researchers to perform proper controls and/or replicates and the failure of reviewers to request this information.

Currently, there is clearly a need for comprehensive reporting of experimental conditions, not only to improve reproducibility but also to reduce the cost of Western blotting. As more researchers report antibody catalog numbers and blotting conditions, better antibodies will be identified and used more often, while poor quality antibodies will be used less frequently, and other researchers will be able to utilize protocols that work well for specific antibodies.

In this article we provide experimental evidence for the need of a Western blotting minimal reporting standard (WBMRS) in all publications using Western blotting. The new information will only add about 100 words to a manuscript but will have a tremendous impact on improving Western blot analysis. Since a few previous reports have suggested that commonly used antibodies give artifactual Western blotting signals [4, 5] we hypothesized that inaccuracies in Western blot analysis would be more prominent with antibodies to more complex epitopes such as post-translational modifications (PTMs). Our investigation suggests that the problem of artefactual signals observed with some antibodies directed against a single target is also observed when using antibodies directed against an epitope found on multiple targets (such as ubiquitination and ISGylation). Experimental data also suggest that besides the quality of the antibody, other commonly modified steps used in Western blotting also significantly alter the results obtained. Overall our results strongly suggest that there is an urgent need for comprehensive reporting of experimental conditions to improve Western blot analysis.

## Materials and Methods

### Materials

Urea, DTT, and buffer reagents were purchased from Sigma-Aldrich (St. Louis, MO, USA). Purified ubiquitin (BML-UW8795), ubiquitin chains (BML-UW0825), and MG-132 (BML-PI102) were obtained from Enzo Life Sciences (NY, USA).

### Cell Culture

H9c2 rat cardiac cells (ATCC, CRL-1446) were grown in DMEM (Invitrogen, Carlsbad, CA) in the presence of 5% FBS and 0.5% Penicillin/Streptomycin (Invitrogen) in a humidified incubator at 37°C and 5% CO_2_. The proteasome inhibitor MG132 (10μM final concentration) was added to the growth media and samples collected 36 h after treatment.

### Sample Preparation

Male mice were euthanized at 3 months of age by inhalation of 3% isoflurane and subsequent cervical dislocation. Hearts were removed, washed in cold PBS, weighed, and flash frozen. This method has been demonstrated to be excellent for investigation by Western blotting [16]. Male Fisher 344-Brown Norway rats (young (10 month old) and old (30 month old)) were assigned to one of two experimental groups: normal or hindlimb-suspension groups. It has been previously shown that 14 days of hindlimb suspension increases the predisposition of rats to get cardiac arrhythmias, while 21 days of hindlimb suspension significantly decreases the turnover rates of cardiac muscle proteins [17, 18]. It is possible that ISGylation would be affected in hearts from hindlimb suspended animals since some of the enzymes that promote ISGylation are also involved in ubiquitin conjugation [19]. To determine if the levels of ISGylated proteins (proteins which are covalently linked to ISG15) in hearts were affected by unloading of the lower limb muscles the noninvasive tail suspension model was utilized [20]. The rats were maintained in head-down tilt position with their hindlimbs suspended for 14 days. Hearts were collected after rats were anesthetized with isoflurane gas. After tissue removal was completed, the rats were killed by exsanguination. Hearts were weighed, frozen in liquid nitrogen, and stored at -80°C for later analysis. This investigation was carried out in strict accordance with the recommendations in the Guide for the Care and Use of Laboratory Animals of the National Institutes of Health. All animal procedures were approved by the University of California, Davis Institutional Animal Care and Use Committee and all efforts were made to minimize suffering.

### Preparation of Purified Polyubiquitinated Proteins and Lysate without Ubiquinated Proteins

Rat cardiac H9c2 cells were exposed to 10μM MG-132 (a proteasome inhibitor) for 36 h to drastically increase the levels of ubiquinated proteins. Samples were then sonicated in 50 mM Tris-HCl, pH 7.5, 0.15 M NaCl, 1 mM EDTA, 1% NP-40, 10% glycerol, and protease inhibitors (P2714, Sigma) at 4°C. After centrifugation (14,000xg) for 10 min at 4°C, the supernatant was incubated with Tandem Ubiquitin Binding Entities (TUBEs) bound to agarose (TUBE2-agarose) (UM402, obtained from LifeSensors, PA, USA). TUBEs display a significantly higher increase in affinity for polyubiquitin moieties (up to 1000-fold) over the single ubiquitin binding associated domain (UBA) [21]. For every mg of total protein, 25 μl of resin was utilized and incubated for 1 h at 4°C with rocking. TUBE-agarose was collected by low speed centrifugation (1000xg, 4°C) for 2 min. The beads were washed with Tris-buffered saline containing 0.05% Tween-20 (TBST) and collected by low speed centrifugation. Washing was repeated three times and the polyubiquinated proteins eluted with 0.2 M glycine HCl, pH 2.5 (3X total volume of pelleted resin) for one 1 h at 4°C. Beads were removed by centrifugation at 10,000xg for 5 min. The eluted polyubiquitinated proteins were quantified, mixed with Laemmli sample buffer and heated at 95°C for 5 min.

### Preparation of Cytosolic Cardiac and Liver Homogenates

Pulverized rat heart or chopped mouse heart or rat liver tissue was homogenized in cold homogenization buffer (50 mM Tris, 1 mM EDTA, 150 mM NaCl, 5 mM MgCl_2_, 0.5 mM DTT, pH 7.5) with a glass dounce homogenizer (25 strokes), and the homogenates were centrifuged at 12,000xg and 4°C for 30 min. The supernatants were removed, diluted to equal protein concentrations, combined with 4X SDS sample buffer (8% SDS, 40% glycerol, 0.4% bromophenol blue, 5% β-mercaptoethanol, 240mM Tris, pH 6.8), and boiled 4 min at 95°C.

### Protein Concentration Determination

The protein concentrations of cytosolic cardiac homogenates were determined with a Nanodrop 2000c (Thermo Scientific). The Nanodrop is the preferred method for determining the concentrations of cytosolic fractions since nucleic acid contamination, which affects the absorbance values on a Nanodrop, is usually absent in these samples.

### Western Blot Analysis

Protein samples (20 μg/well) and dual plus molecular weight ladders (#161–0374, Bio-Rad Laboratories, Hercules, CA) were separated by SDS-PAGE on Criterion Stain-free and conventional Precast Gels with a 4–15% gradient (Bio-Rad) for approximately 60 min at 150V in running buffer (25 mM Tris base, 192 mM glycine, 1% SDS, pH 8.3). Stain-free gels were activated by exposure to UV for 1 min. Proteins were transferred to nitrocellulose membranes (#170–4159, Bio-Rad) using the Bio-Rad Trans-Blot Turbo Transfer System for 7 min. Total proteins on membranes were detected using the Stain-free method [22, 23] or with ponceau S staining. All Western blotting procedures were carried out at room temperature with agitation except when stated otherwise. Membranes were blocked with 3% non-fat milk (# 170–6404 Bio-Rad) in TBST (20 mM Tris, 150 mM NaCl, containing 0.05% Tween-20, pH 7.4) or PBST (10 mM phosphate, 137 mM NaCl, 2.7 mM KCl, containing 0.05% Tween-20, pH 7.4) for 60 min. Membranes were then incubated with primary antibodies (see Table 1) in TBST or PBST with 1% non-fat milk at room temperature for 2 h or at 4°C overnight. Removal of excess primary antibody was carried out by washing the membranes in TBST or PBST three times for 5 min each. The secondary antibody (peroxidase-conjugated anti-mouse, anti-goat, or anti-rabbit IgG secondary antibody (anti-mouse Cat. # A9044, anti-goat #A5420, anti-rabbit Cat. #A0545, Sigma-Aldrich, St. Louis, MO, USA)) diluted 1:5000 was incubated with the membrane in TBST or PBST with 1% non-fat dry milk for one h at room temperature. Excess secondary antibody was removed by washing the membranes in TBST or PBST three times for 5 min each. Membranes were exposed to Clarity enhanced chemiluminescence (ECL) reagent (Cat. # 170–5061, Bio-Rad) for 2 min at room temperature and visualized using a ChemiDoc MP (Cat. # 170–8280, Bio-Rad). Detection and quantification of band intensities was conducted using Image Lab 5.0 software (Bio-Rad). Bands were normalized to total protein by dividing the intensity of the band by the intensity of the total protein from the same sample on the same blot. Background correction was carried out as described by Taylor et al. [24]. At least three biological replicates were used for young and old rat studies while at least three technical replicates were used for the heart and liver comparisons.

**Table 1:** Table showing the primary antibodies used and basic information about these antibodies.

Antibody Target	Antibody Name/ catalog #/ lot number	Supplier	Type of Antibody/concentration of antibody as reported by manufacturer	Citations on manufactur-er’s website/ citations on CiteAb *
Ubiquitin	VU1-ubiquitin/VU101	LifeSensors	Monoclonal/0.5mg/mL in PBS	0/0
	Ubiquitin/U5379/089K6001	Sigma	Rabbit Polyclonal/NR	26/36
	Ubiquitin/AP1228a/RB0643	Abgent	Rabbit Polyclonal/NR	1/1
	P4G7-H11/ADI-SPA-203-D	Enzo	Monoclonal/NR	1/0
	FK1/PW8805	Enzo	Monoclonal/0.5mg/mL in PBS	24/9
ISG15	H150/sc-50366/A0509	Santa Cruz	Rabbit polyclonal/ 200μg/mL in PBS	8/7
	R140/sc-50368/E0107	Santa Cruz	Rabbit polyclonal/ 200μg/mL in PBS	0/1
	M20/sc-18421/B0613	Santa Cruz	Goat polyclonal/ 200μg/mL in PBS	0/0
	F9/sc-166755	Santa Cruz	Monoclonal/200μg/mL in PBS	0/0
	E9/sc-166794	Santa Cruz	Monoclonal/200μg/mL in PBS	0/0
	ISG15/14-5857	EBioscience	Monoclonal/500μg/mL in aqueous solution	5/5
β-Actin	β-Actin/sc-47778	Santa Cruz	Monoclonal/200μg/mL in PBS	565/715
PSMA6	PSMA6/ab109377/YH120115	Abcam	Rabbit Monoclonal/ NR	1/1

NR, Not reported.

* Number of publications using these antibodies that are listed on the manufacturer’s website and on CiteAb website as of July 1^st^ 2015.

### Statistics

The results are shown as mean ± standard deviation (SD). Statistical significance was determined by student’s t-test or one-way ANOVA. Comparisons yielding a value of p < 0.05 were regarded as statistically significant.

## Results and Discussion

Western blotting is a powerful technique for quantifying protein levels; however it is often not well optimized and relies greatly on antibodies which are poorly validated. As our study suggests, antibodies differ in regards to the optimal blotting conditions and results they yield; even different antibodies to the same target peptide can give different results. Currently more polyclonal antibodies are used for Western blotting than monoclonal antibodies, mainly due to the ease and lower up-front cost of making polyclonal antibodies. However, polyclonal antibodies vary from lot to lot due to different animals, improper storage, and different bleeds from an individual animal. Since the previously mentioned studies in S1 Table as well as other studies have shown that some popular antibodies to specific proteins show artifactual signals [3–5], we investigated two sets of antibodies to common PTM epitopes associated with ISGylated and ubiquitinated proteins.

### Comparison of Ubiquitin Antibodies

Ubiquitin antibodies have been developed to target free ubiquitin, ubiquitin chains linked in a specific manner, or ubiquitin in any form. Depending on the quality and specificity of the ubiquitin antibody used, different researchers may obtain different results when examining ubiquitination or free ubiquitin levels. Our objective was to compare ubiquitin blots using five different antibodies to see if they gave similar results. It was expected that most anti-ubiquitin antibodies would detect high molecular weight polyubiquitinated proteins in the heart and liver samples as well as the polyubiquitinated protein-enriched lysate. It was also expected that the main polyubiquinated proteins detected would be similar to other anti-ubiquitin antibodies.

**Fig 1:**
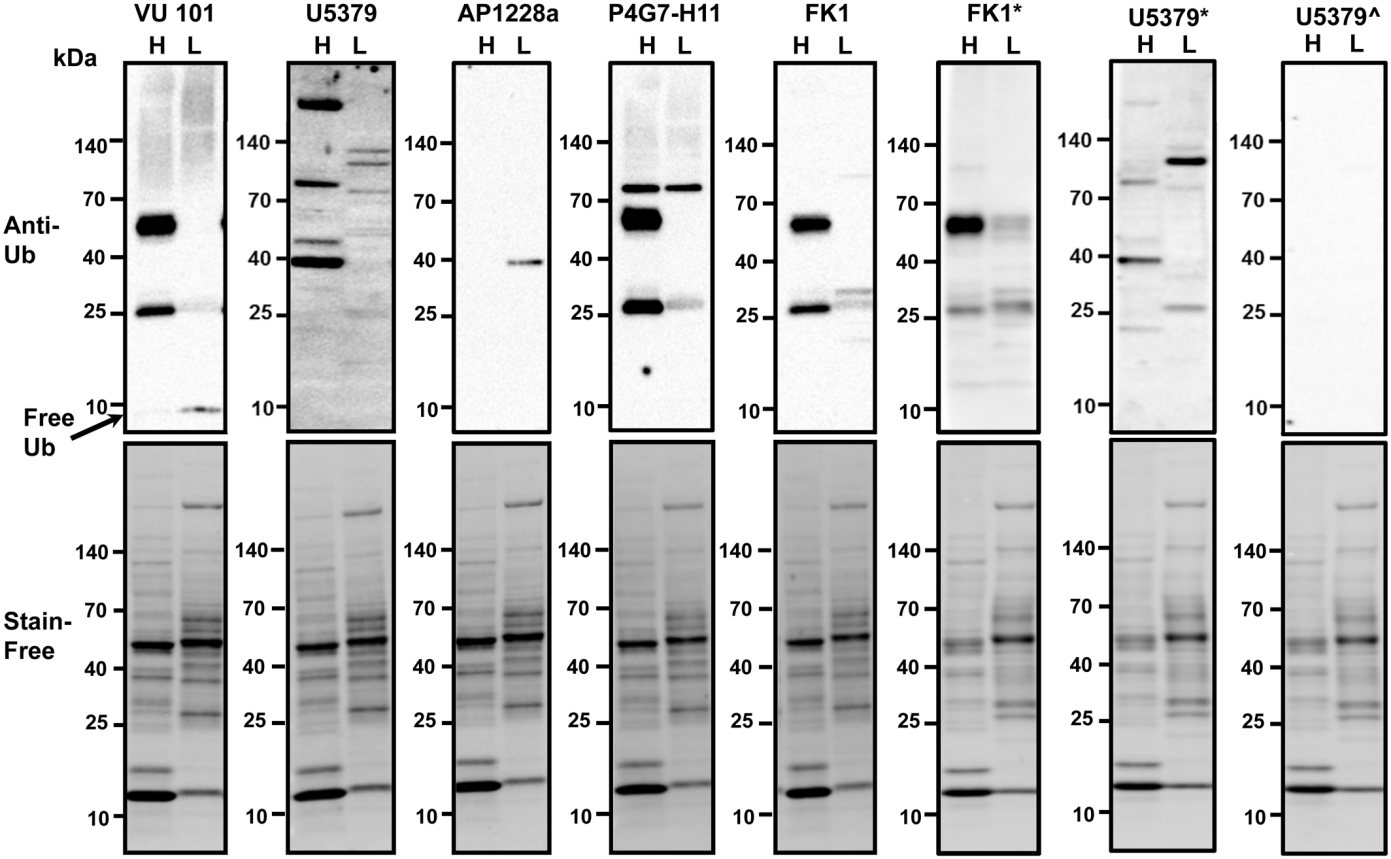
Comparison of anti-ubiquitin antibodies. Heart and liver lysates (20 μg each) were investigated by Western blotting using five commercially available anti-ubiquitin antibodies (VU101, U5379, AP1228a, P4G7-H11, FK1). Arrow shows location of free unbound ubiquitin. Stain-free staining of total proteins loaded was used as the normalization control. H, heart; L, liver. BSA was used as the blocking reagent for the blot labeled FK1* while non-fat milk was used as the blocking reagent in all the other blots shown. All antibodies were used at a dilution of 1:1000 except for blots labeled U5379* and U5379^ which were used at dilutions of 1:100 and 1:2000 respectively.

Comparison of the mouse heart and liver cytosolic fractions (20 μg each) using five commercially available antibodies showed that three antibodies identified consistent major bands at approximately 26 and 60 kDa (Fig 1). These antibodies were utilized under the same conditions (1:1000 dilution) except for the last two lanes on the right which were at 1:100 and 1:2000 dilutions (Fig 1). Two antibodies (VU101 and P4G7-H11) detected a large number of high-molecular weight ubiquitinated proteins while another antibody (U5379) detected some high molecular weight bands. However, only one antibody (VU101) detected free ubiquitin in the liver samples under the conditions investigated (the location of free ubiquitin is shown by an arrow in Fig 1). Four antibodies showed higher levels of ubiquinated proteins in heart than liver for the same amount of total protein. The antibody that did not show more ubiquitination in heart (AP1228a) detected no proteins in the heart sample and only one protein in the liver sample. Although two antibodies, VU101 and FK1, gave similar results, the FK1 antibody did not detect high molecular weight proteins. Since the manufacturer recommends the FK1 antibody be utilized in BSA instead of nonfat milk (NFM), Western blot analysis using FK1 was also carried out using 1% BSA in TBST (lane FK1* in Fig 1). All the other blots were carried out using NFM. When BSA was used instead of NFM a few additional bands were detected.

Since antibody concentration is also important, all antibodies were utilized at the dilution that was recommended by the manufacturer. The antibodies VU101, AP1228a, P4G-H11, and FK1 are all recommended for use at 1:1000 while U5379 is recommended at a concentration of 1:100. Varying the concentration of U5379 from 1:100 to 1:2000 showed the importance of the concentration of antibody used, as the 1:2000 dilution only faintly detected one high molecular weight band in the liver sample. However, independent of blocking reagent used or concentration of antibody, the results suggest that different anti-ubiquitin antibodies give distinctly different banding patterns when using Western blot analysis.

**Fig 2:**
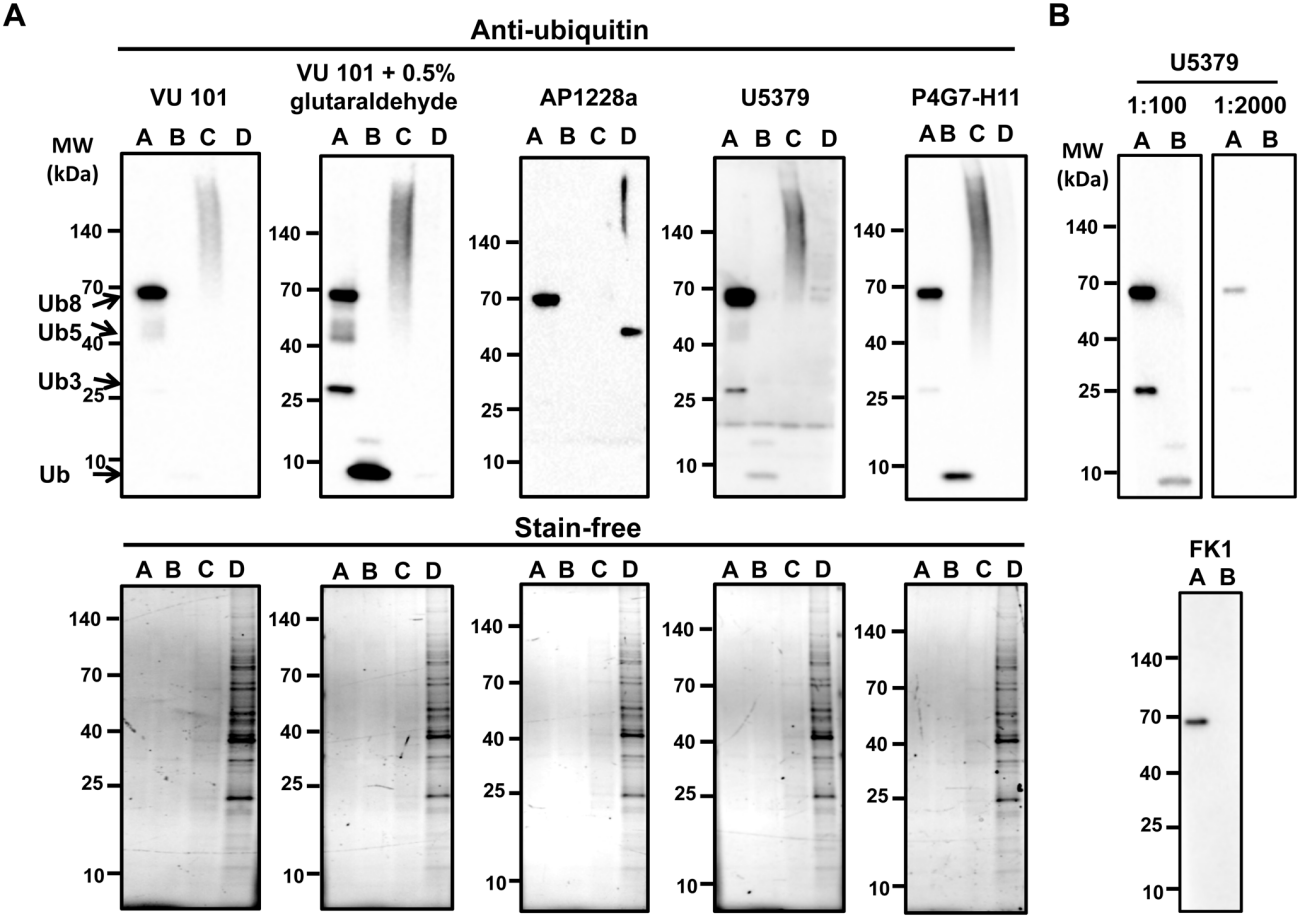
Validation of anti-ubiquitin antibodies. VU101 in the presence and absence of 0.5% glutaraldehyde pre-treatment, U5379, AP1228a, or P4G7-H11 were used to detect ubiquitin and ubiquitinated proteins. A) Western blot of polyubiquitin chains (Ub3, Ub5, Ub8) (lane A), purified ubiquitin (lane B), polyubiquitinated proteins from H9c2 cells treated with 10μM MG-132 for 36 h obtained from affinity purification using TUBEs (lane C), and unbound fraction from H9c2 cells after removal of polyubiquitinated proteins (lane D). B) Upper figure, Western blot of free ubiquitin (lane A) and polyubiquitin chains (lane B) with U5379 antibody diluted at 1:100 and 1:2000. Lower figure, Western blot of free ubiquitin (lane A) and polyubiquitin chains (lane B) with FK1 antibody diluted at 1:1000 in BSA. Even when the blots were imaged for long time periods no additional bands were seen.

Further validation of these antibodies showed that one of these antibodies (AP1228a) did not recognize either free ubiquitin or polyubiquitinated proteins (Fig 2). FK1 only recognized one of the polyubiquitin chains and did not recognize free ubiquitin under the conditions utilized (Fig 2). Purified ubiquitin was used as the positive control for free ubiquitin, while commercially obtained polyubiquitin chains (tri-ubiquitin, penta-ubiquitin and octa-ubiquitin chains) were used as a positive control for polyubiquitin chains. Purified polyubiquitinated proteins and lysate depleted of polyubiquitinated proteins were made in our laboratory using TUBEs. TUBEs has been shown to be highly efficient at removing polyubiquinated proteins from lysates [25]. Two steps were taken to ensure the lysate was depleted of ubiquitinated proteins: significantly more bait (TUBEs) was used than required, and the lysate remaining after the ubiquinated proteins were removed was further processed with TUBEs to remove any trace amounts of ubiquinated proteins. Use of the different controls showed that the VU101 antibody was the best antibody for detection of free ubiquitin and polyubiquitinated proteins.

Due to the relatively low amounts of polyubiquitin chains (300 ng) and purified ubiquitin (1 μg) used, no protein bands were detectable by total protein staining methods such as Stain-free (Fig 2). Stain-free works by cross-linking a fluorescent adduct to tryptophan residues, so the Stain-free method will not detect the tryptophan-less ubiquitin [22]. The VU101 antibody was able to detect all three polyubiquitin chains, the purified ubiquitin, and numerous proteins in the purified polyubiquitinated samples, while detecting only free ubiquitin in the polyubiquinated protein depleted samples. TUBEs do not bind free ubiquitin with high affinity so free ubiquitin was expected to be present in the polyubiquinated protein-depleted fractions. In the experiments shown in Fig 1 the VU101 was used without glutaraldehyde pretreatment. The manufacturer’s protocol for VU101 suggests for optimal results the membrane should be pre-treated with 0.5% glutaraldehyde (Fig 2). Although VU101 recognized all the positive controls without pre-treatment, inclusion of the 0.5% glutaraldehyde pre-treatment increased the signal intensity of free ubiquitin and polyubiquitinated proteins. Inclusion of 0.5% glutaraldehyde pre-treatment in the protocol for Western blotting using other anti-ubiquitin antibodies resulted in no bands being detected, suggesting that the pre-treatment affects the antibody-antigen interactions (data not shown).

For both VU101 and U5379, ubiquitin dimers were also detected. Non-covalent dimerization of free ubiquitin has been previously described [26]. Of concern is that the AP1228a antibody detected a major band in the lysates from which polyubiquitinated proteins were removed. While this antibody detected the octa-ubiquitin chain it did not detect the tri- and penta-ubiquitin chains, the polyubiquitinated proteins in the polyubiquitinated enriched lysate, or free ubiquitin. It is possible that the protein detected by AP1228a is a monoubiquitinated protein still present in the polyubiquitinated depleted lysate. P4G7 detected two of the polyubiquitinated chains, free ubiquitin and polyubiquitinated proteins in the polyubiquitinated enriched lysate, suggesting that it is also a good antibody with the limitation that it does not recognize all polyubiquinated chains. The U5379 showed reactivity to two of the polyubiquinated chains, but also detected some proteins in the polyubiquinated depleted lysate. The VU101, which was the best anti-ubiquitin antibody according to our results, had fewer citations than four of the other anti-ubiquitin antibodies (Table 1). These results suggest that using different antibodies to examine ubiquitination may give contradicting results, as each antibody recognized different subsets of proteins, with some anti-ubiquitin antibodies not recognizing polyubiquitinated standards and at least one antibody recognizing a potentially non-ubiquitinated target. Interestingly, the most commonly cited antibody in CiteAb is the anti-ubiquitin antibody U5379 from Sigma Chemical Company, while the best performing antibody VU101 had no references on CiteAb. The results from four of the antibodies tested suggest that 20 μg of mouse heart contains more polyubiquinated proteins than 20 μg of mouse liver. The anti-ubiquitin antibody U5379 showed distinct target binding properties compared to any of the other anti-ubiquitin antibodies. VU101, P4G7-H11 and FK1 antibodies all detected two major polyubiquitinated proteins at 25 and 58 kDa while the U5379 antibody did not detect either of these bands (Fig 1). While the U5379 antibody clearly detects polyubiquinated proteins (Fig 2), this antibody also detected proteins in the polyubiquinated protein depleted lysate suggesting that this antibody could potentially be recognizing non-ubiquitinated proteins. It is also possible that U5379 may be detecting monoubiquinated proteins in the polyubiquinated protein depleted lysate. The potential detection of some non-ubiquitinated proteins may account for the significantly different target protein identification obtained with this antibody compared to the other antibodies. A possibility also exists that the tri-, penta, and octa-polyubiquinated chains may be forming dimers which would further complicate the analysis of the Western blots. These results emphasize that the most popular antibody is not necessarily the best antibody for the target protein(s).

Based upon the results obtained it is recommended that positive controls should be included when Western blot analysis is carried out using anti-ubiquitin antibodies. It is in the interest of the scientists working in this field to establish what the best optimal controls would be.

### Comparison of ISG15 Antibodies

**Fig 3:**
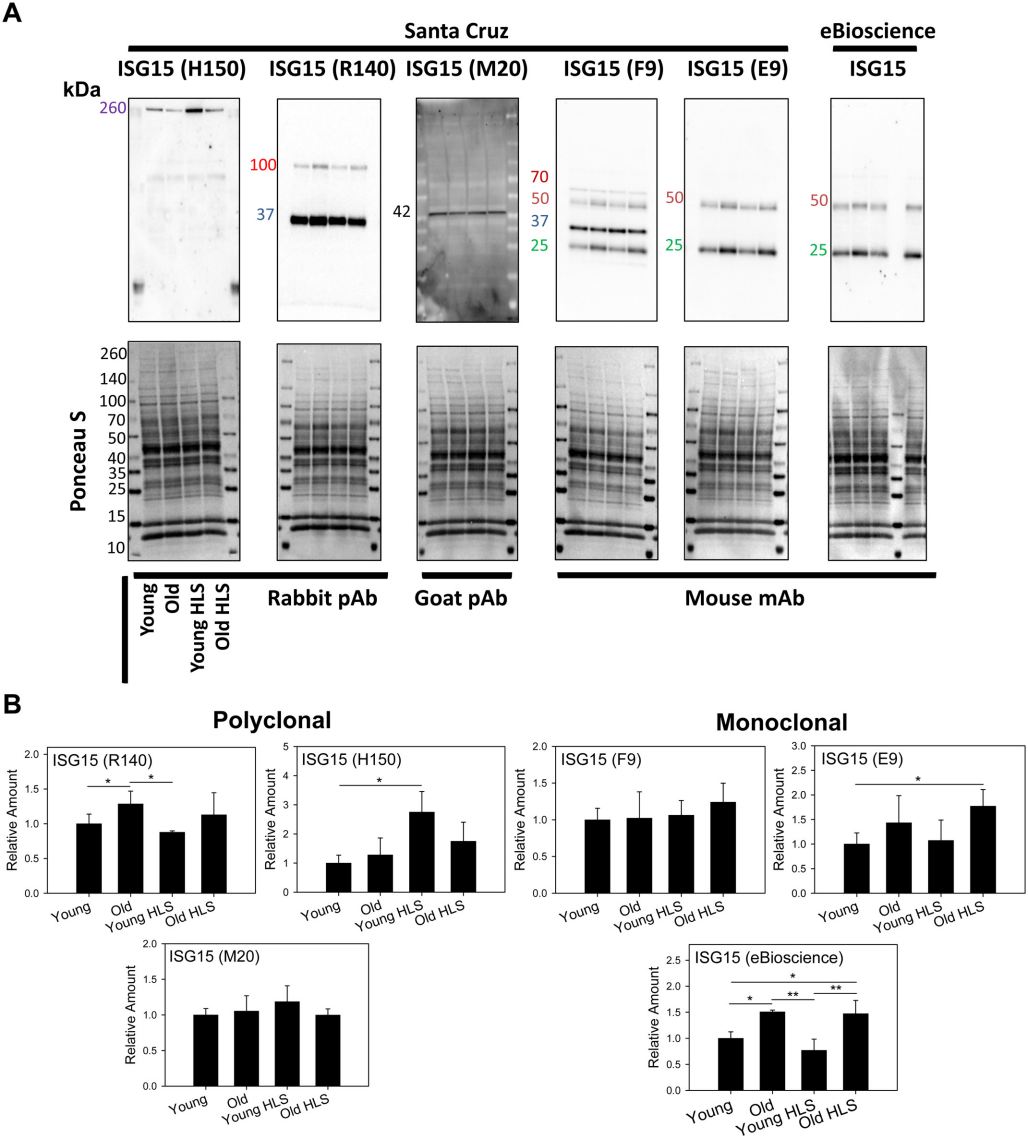
Comparison of anti-ISG15 antibodies. (A) Seven anti-ISG-15 antibodies were used to detect the levels of ISGylated proteins in four different types of samples. (B) Quantification of ISG15 Western blots. Young, 10 month old hearts; Young HLS, high-limb suspended 10 month old hearts; old, 30 month old hearts; Old HLS, high-limb suspended 30 month old hearts. * p < 0.05, ** p < 0.01 by 1-way ANOVA.

ISG15 is another small protein modifier that can be conjugated to proteins to regulate their activity. Proteins which are covalently linked to ISG15 are referred to as ISGylated proteins. The effect of aging or skeletal muscle disuse on ISGylated protein levels in hearts has not been previously reported. To investigate this, we initially utilized two antibodies against ISG15 and expected to find that one antibody would detect more ISGylated proteins than the other antibody but that both antibodies would detect the same main ISGylated proteins. However, we obtained significantly different results for the two antibodies by Western blot analysis. Further investigation of five anti-ISG15 antibodies from Santa Cruz and one from eBioscience showed that only two of these antibodies gave similar results (Fig 3). E9 and ISG15 antibodies from Santa Cruz and eBioscience respectively (both monoclonal) gave similar results. The samples that were investigated were young and old hearts from normal (control) and hind-limb suspended (HLS) rats. The most common major bands recognized in these samples were 25 and 50 kDa bands which were identified by three antibodies tested. The other most common bands were 37, 42, and 100 kDa which were recognized by two antibodies each. The H150 antibody was the only antibody that recognized a 260kDa protein band. This H150 antibody which gave different results from every other anti-ISG antibody investigated is currently the most cited anti-ISG15 antibody (Table 1). Validation of these ISG15 antibodies was not carried out, because we were unable to enrich for ISGylated proteins without using antibodies, and ISG15 siRNA was only able to reduce ISG15 levels by 80% in C2C12 skeletal muscle cells. Quantification of the results to determine if old rat hearts have similar, lower, or higher levels of ISGylated proteins than young hearts showed that three antibodies detected higher levels of ISGylated proteins in old hearts when compared to young hearts, while four other antibodies showed no statistically significant change (Fig 3B). One antibody (H150) showed increased ISGylated protein levels in young HLS hearts when compared to young hearts while other antibodies showed similar ISGylated protein levels in these hearts. The eBioscience anti-ISG15 antibody showed higher ISGylated protein levels in old HLS hearts when compared to young HLS hearts, while the other six antibodies showed similar protein levels in HLS hearts. These results strongly suggest that different ISG15 antibodies recognize different epitopes and give different results.

The results of our investigation of anti-ISG15 antibodies were more complicated than the ubiquination results, as five of the six antibodies detected different proteins. The most cited anti-ISG15 antibody on CiteAb was H150, and the second most cited was anti-ISG15 from eBioscience. Both of these antibodies give distinctively different banding patterns. Bands at 37 and 100 kDa detected by at least two other antibodies were not detected by either H150 or eBioscience anti-ISG15. The H150 antibody showed a >2.5 fold increase in ISGylated protein levels (mainly due to a 260kDa protein) in young HLS hearts when compared to young hearts while the anti-ISG15 from eBioscience showed no such change. These results show that depending on the ISG15 antibody used, different results can be obtained. Hence it is critical to have the catalog number of the antibody used in all publications. The ratio of sub-standard antibodies to quality antibodies is likely to increase, as the number of post-translational modification (PTM) specific antibodies is increasing at an exponential rate. Antibodies are now available for many PTM sites including acetylation, methylation, and the more common phosphorylation sites, but many of these PTM specific antibodies do not work well. An additional complication is that the antibody specificity may change under different experimental conditions and in different tissues [27]. An antibody may work well for one cell type or species but not for other cell types.

Even if the scientific community is able to generate quality monoclonal antibodies or recombinant antibodies for Western blotting, other factors important for the Western blotting technique also need to be taken into account. One example is the primary antibody concentration. The amount of antibody that should be utilized for Western blotting depends on many factors including the concentration of the enzyme, the abundance of the target protein and the detection system used. The specificity and high affinity of antibodies for their targets allows antibodies to be used in low concentrations (ranging from 1:100 to 1:500,000 for a 1mg/ml starting concentration). The optimal dilution of the primary antibody has to be determined experimentally. In general lower amounts of antibody result in increased specificity for the target protein. Using lower amounts of antibody also reduces cost.

Overall, similar to the anti-ubiquitin antibodies, proper Western blot analysis using anti-ISG15 requires proper controls. An ISG15 knock-out mouse is available and tissues from this mouse model would be an excellent negative control for anti-ISG15 antibodies. It may be time for NIH to start a resource center with tissues from knock-out animal models that could be used as negative controls for Western blot analysis. Including information in manuscripts about controls or validations done using antibodies not previously validated would give reviewers greater confidence in the results and would also be beneficial to other researchers [2, 28].

### Effect of Primary Antibody Dilution on Western Blotting Results

**Fig 4:**
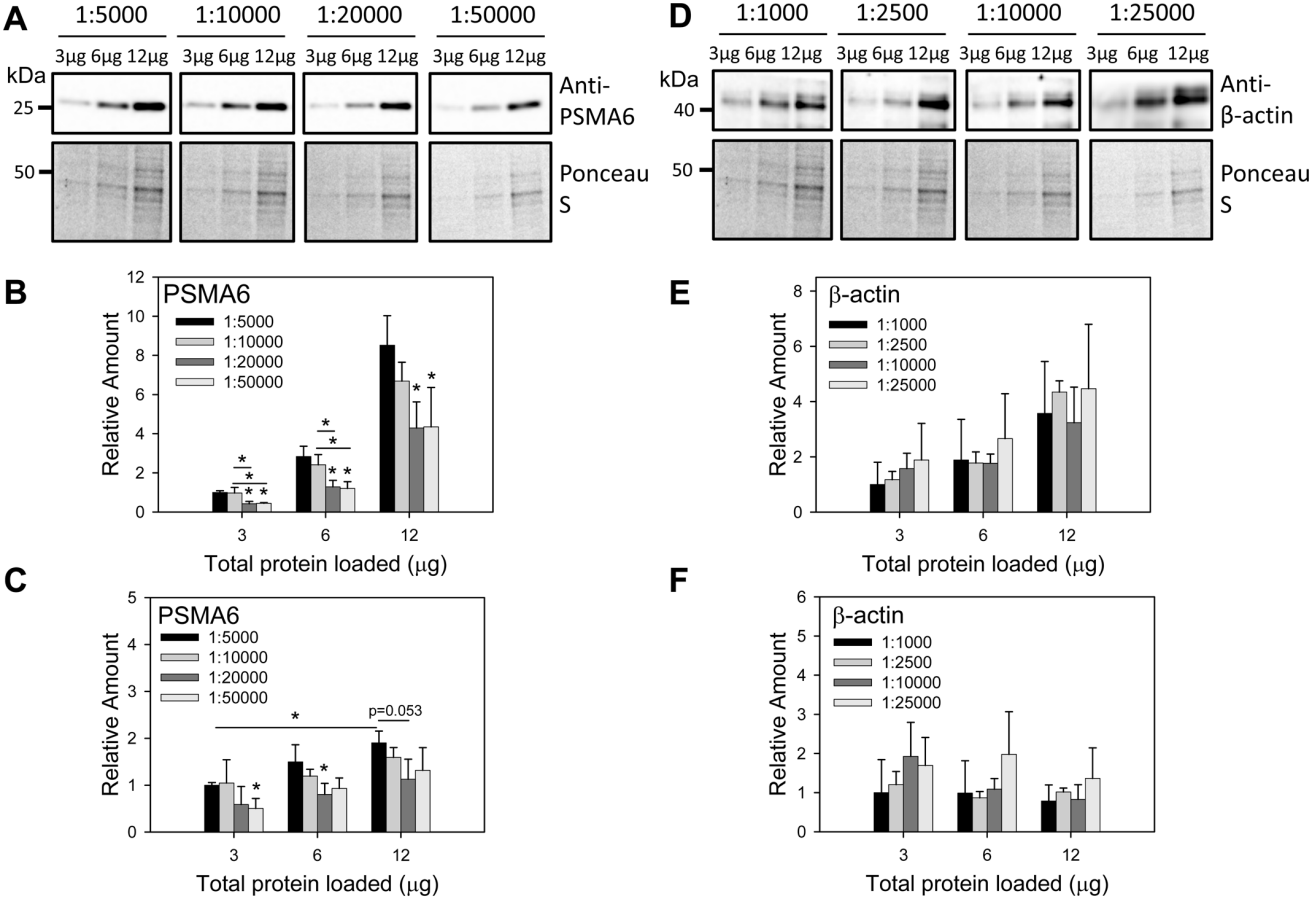
Effect of antibody concentration on linearity of target proteins detected by Western blotting. (A) Western blot of rat liver samples (3–12 μg) using anti-PSMA6 at four different concentrations (1:5000, 1:10000, 1:20000, and 1:50000). (B) Quantification of anti-PSMA6 Western blots without including any normalization. (C) Quantification of anti-PSMA6 Western blots using total protein normalization. (D) Western blot of rat liver samples (3–12μg) using anti-β-actin at four different concentrations (1:1000, 1:2500, 1:10000, and 1:25000). (E) Quantification of anti-β-actin Western blots without including any normalization. (F) Quantification of anti-β-actin Western blots using total protein normalization. * p < 0.05 by 1-way ANOVA.

Since other factors are also important for Western blotting accuracy and reproducibility, we investigated two of these important factors which are often overlooked: 1) the effect of primary antibody dilution on signal detected and 2) the effect of the main buffer used in Western blotting on signal detection. Different concentrations of rat liver lysates were probed with different dilutions of anti-β-actin and anti-PSMA6 to determine the effect of antibody dilution on signal detection. β-actin is a commonly used Western blotting normalization control and PSMA6 is a subunit of the proteasome. While it was expected that lower concentrations of antibody (higher dilutions) would result in lower signal intensity, we observed that anti-β-actin dilution did not significantly affect the signal intensity detected (Fig 4E). This was surprising since a 1:25000 dilution of the anti-β-actin gave similar results to a 1:1000 dilution. Using this 25 fold dilution of anti-β-actin would significantly reduce the cost of the Western blot without compromising the sensitivity of detection.

In contrast, higher concentrations of anti-PSMA6 (1:5000) gave higher signal intensities of PSMA6 when compared to lower concentrations of the anti-PSMA6 at all liver lysate amounts investigated (Fig 4B, not normalized data). When normalized to total protein loaded it is expected that all the protein concentrations and antibody dilutions would give a normalized value of 1 as observed for β-actin (Fig 4F). However lower concentrations of anti-PSMA6 resulted in lower normalized signal intensities at 3 and 6 μg of liver lysate (Fig 4C). Also of significance is that the normalized values for 12μg of lysate was statistically significantly higher than for 3μg of lysate when the anti-PSMA6 was used at its highest concentration (1:5000 dilution, Fig 4C). This suggests that to achieve optimal results with antibodies such as the anti-PSMA6 antibody used in these studies (as opposed to the β-actin antibody used in these studies), it is important that the amounts of proteins loaded on a gel be similar in each lane. In contrast to the PSMA6 antibody, using higher amounts of the β-actin antibody did not increase the signal intensity, suggesting that for certain antibodies it is not advantageous to use higher amounts of antibody (Fig 4E). These results show that the amount of protein loaded on a gel is important, and each gel lane should have very similar total protein amounts to avoid artefacts due to protein loading.

#### Effect of buffer on western blotting results

**Fig 5:**
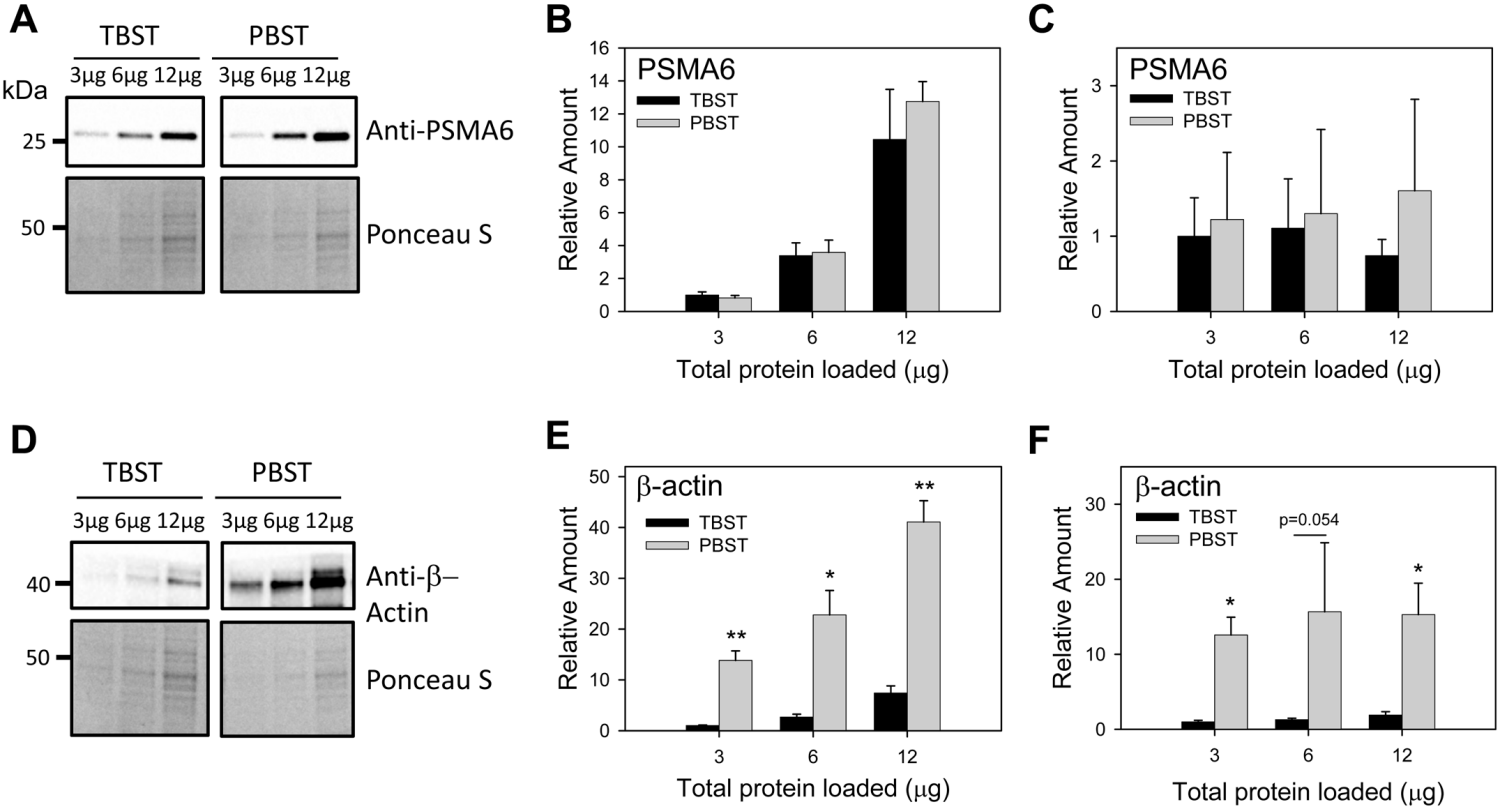
Effect of buffer reagent on Western blotting linearity. (A) Western blot of rat liver samples (3–12 μg) using anti-PSMA6 and different buffers (TBST and PBST). (B) PSMA6 quantification, not normalized to total protein. (C) PSMA6 quantification, normalized to total protein. (D) Western blot of rat liver samples (3–12 μg) using anti-β-actin and different buffers. (E) β-actin quantification, not normalized to total protein. (F) β-actin quantification, normalized to total protein. * p < 0.05, ** p < 0.01 by 1-way ANOVA.

A variety of Western blotting buffers are currently used and range from TBS or PBS without any additives to TBS and PBS with several additives including Tween-20 and low amounts of blocking reagent such as non-fat milk. TBS, however, a commonly used buffer for Western blots, is not always the best buffer for certain antibodies. Commercial preparations of TBS can also be different in the concentration of Tris present and the pH of the solution. We carried out a Western blot on rat liver lysates with the PSMA6 and β-actin antibodies using TBST or PBST to see if varying the buffer affected our results (Fig 5). Although some researchers utilize PBS while other utilize TBS we expected that these buffers would result in different target protein signal intensities. While anti-PSMA6 antibody showed similar normalized relative amounts of detected targets in different amounts of rat liver lysates (3–12μg), anti–β-actin showed a greater than 10 fold increase in intensity when PBS was used instead of TBS. When low abundance targets are being detected, 10 fold differences in the detection signal could be the difference between detecting the protein of interest and not detecting the protein. These results show that the choice of buffer is very important for signal intensity when certain antibodies are used. It is recommended that Western blot analysis using uncharacterized antibodies be carried out with both PBS and TBS to determine if one buffer is significantly superior to the other buffer.

### Other Western Blotting Concerns

Several other common Western blotting problems exist; however since experimental data from different laboratories including our laboratory are already available on this topic these problems are only briefly mentioned [29–35]. A common problem in Western blotting is the total amount of protein in each well is too high. When a protein of interest is expressed at very low amounts, some labs use very high protein loads (>60μg of total protein) to be able to detect the protein and use a housekeeping protein (which is usually expressed at relatively high levels) as a loading control. While the protein of interest may be in the dynamic range of the tissue or sample being investigated, housekeeping proteins because of their abundance have a limited dynamic range and are not linear at high protein concentrations [33]. Another problem with blotting high amounts of proteins is the increased chance of artifacts due to non-specific binding of the antibody utilized. Using lower amounts of protein for Western blotting has been shown to improve the detection of some poorly expressed proteins [30, 31]. The secondary antibody used also affects the signal intensity of the target protein. IgG subclass specific secondary antibodies were found to be superior to anti-mouse IgG (H+L) antibodies in immunohistochemistry and Western blotting [36]. A recent publication also showed results which demonstrated that the current lack of established procedures in densitometry results in inaccurate quantification of many Westerns [34].

All of these reports suggest that the current standards for reporting Western blots are inadequate. A requirement by journals for researchers to deposit detailed antibody information into one of the online databases would be very beneficial, but the best solution would be an NIH mandated depositing of detailed Western blotting data into one site for all NIH funded research. A repository with the tissue used and optimized buffer for antibodies would be especially useful to all research labs using this technique. Like polymerase chain reaction and mass spectrometry data, reporting minimal standards are needed for Western blotting. A 10 point requirement referred to as the Western blotting minimal reporting standard (WBMRS) is suggested (Table 2 and S1 text). With limited research funding, identifying poor quality antibodies and poor Western blotting techniques will save money, save researchers time and improve the quality of the results.

**Table 2:** Western Blotting Minimal Reporting Standard (WBMRS).

Point	Needed	Reason
1)	The primary and secondary antibodies used, the catalog number, company purchased from, and lot number if it is a polyclonal antibody.	Possibly by recognizing alternatively spliced variants, PTM configurations etc. In a few cases antibodies can recognize non-specific bands around the molecular weight of the target protein. Different antibodies to the same protein can give different results. Currently more polyclonal antibodies are used for Western blotting but vary from lot to lot due to different animals, improper storage, and different bleeds from individual animals.
2)	Molecular mass of band of interest should be shown on the blot.	Several antibodies recognize bands which are not the proper molecular masses of the target protein. In fact some antibodies that are known to recognize only a different molecular weight protein are sold by some companies.
3)	The amount of total protein loaded onto the gel and the method used to determine the protein concentration should be stated.	Use of too much protein results in inaccurate quantification.
4)	Type, amount and extent of use of blocking reagent.	The type and amount of blocking reagents can significantly affect the number of non-specific bands detected by an antibody.
5)	Washing solution used, how often and for how long.	The buffer used and amount of washing affects the background membrane staining and sometimes the number of non-specific bands detected by an antibody.
6)	Amount and incubation time of primary antibody. Primary antibody buffer.	The primary antibody amount and buffer it is diluted in are critical for good Western blots.
7)	Amount and time of incubation of secondary antibodies. Secondary antibody buffer.	The secondary antibody amount and buffer used is important for good Western blots.
8)	How the image was collected.	If X-ray film was used then the source of X-ray film is needed. X-ray film has lower linearity than commercial imagers.
9)	The reagent used for detection and how long the membrane was incubated in the reagent (including company that manufactured the reagent).	Different ECL reagents typically have distinct properties. Different fluorescent secondary antibodies also sometimes have dissimilar properties.
10)	The software (including company that created the program and version number) used for data analysis. Basic information about how the quantification was carried out.	If quantification is incorrectly done then no matter how well the Western blot was carried out the data from the Western blot will be inaccurate.

## Conclusion

Being able to trust the experimental data is critical for experimental research. More detailed Western blotting information will allow some experienced reviewers to detect Western blotting problems before manuscripts are published. Having access to more detailed Western blotting methods is also critical for future advancements since the amount of poor quality Western data in the literature is likely to be understated. Although the suggested WBMRS documentation will allow other researchers to better reproduce and confirm or fail to confirm previously published data, limitations due to experimental error cannot be accounted for. Some parameters which are assumed to be equal in most laboratories are not. A mistake that is sometimes made is to use a blocking solution that contains biotin with a primary or secondary antibody that is covalently linked to biotin. Other examples include incorrect calculations for the amount of antibody, incorrect buffer composition, dirty gel plates and gel boxes, and other components used in Western blotting. Small amounts of SDS or other detergents left on the glass plates used for gel electrophoresis have been shown to increase background [37]. The use of new dishes for washing blots significantly decreases the background in Western blots. However, these are likely to be only a small percentage of Western blots reported. Most scientists have experienced the frustration of purchasing an antibody based on a publication and finding out that the antibody does not work as advertised or that the antibody is unable to detect any proteins. It is recommended that researchers do test blots using different buffers and antibody dilutions to optimize for each Western carried out in the lab. In summary, as a scientific community we will only be able to reproduce and effectively utilize Western blotting data when we have a basic set of reporting standards that allow the commonly used tricks and mistakes to be well documented. Without improvements in the reporting of Western blotting data the increasing number of poor quality antibodies currently available will likely further increase the irreproducibility associated with Western blotting.

## Supporting Information

S1 Table Recent Publications documenting Western blotting inaccuracies with commercially available antibodies. (DOCX)

S1 Text Sample Template for Western blot method incorporating the WBMRS. (DOCX)
