# Supplementary material for: Western Blotting Inaccuracies with Unverified Antibodies: Need for a Western Blotting Minimal Reporting Standard (WBMRS)
Source: PLoS One. 2015 Aug 19;10(8):e0135392. doi: 10.1371/journal.pone.0135392 (PMC4545415; doi:10.1371/journal.pone.0135392)
Supplement: S1 Table — (DOCX) [file pone.0135392.s001.docx]

**S1 Table. Recent Publications documenting Western blotting inaccuracies with commercially available antibodies.**

| Target/Important notes | Antibodies used | Results | Refer-ence |
| --- | --- | --- | --- |
| CCR5 (CD195)/ CCR5 is a membrane associated protein and is present at low levels on human macrophages, making it difficult to detect. | Nine commercially available anti-CCR5 monoclonal antibodies were evaluated for their specificity and their recognition of CCR5 expressed by macrophages. | Three of the nine clones tested displayed substantial background binding to CCR5 negative cells.Conclusion: Several commercially available anti-CCR5 monoclonal antibodies lack specificity and should be used with caution. | [[11](#_ENREF_11)] |
| 57 different histone modifications in *Drosophila melanogaster, Caenorhabditis elegans* and human cells. | > 200 antibodies. | > 25% failed specificity tests by dot blot or western blot.  **Conclusion:** Authors suggest rigorous testing of histone-modification antibodies before use. | [[12](#_ENREF_12)] |
| Angiotensin II AT1 receptor. | Six commercially available Angiotensin II AT1 receptor antibodies. | None of the commercially available AT1 receptor antibodies tested met the criteria for specificity.  **Conclusion:** competitive radioligand binding remains the only reliable approach to study AT1 receptor. | [[7](#_ENREF_7)] |
| GABARAPL1. | Specificity of three antibodies targeted against different peptide sequences within GABARAPL1. | Only one antibody was specific for GABARAPL1 *in vitro* and *in vivo.*  **Conclusion:** It is important to test antibody specificity under the conditions for which it will be used experimentally. | [[27](#_ENREF_27)] |
| Histamine H₄-receptor (H₄R). | Three commercially available H₄R antibodies. | None of the antibodies bound to the specified protein.  **Conclusion:** Importance of evaluation of antibodies using valid controls. | [[8](#_ENREF_8)] |
| Human β3-adrenoceptor. | Five antibodies raised against the full-length protein. | None of the five antibodies exhibited convincing target specificity in immunoblotting.  **Conclusion:** Importance of antibody validation. | [[6](#_ENREF_6)] |
