# Supplementary material for: Western Blotting Inaccuracies with Unverified Antibodies: Need for a Western Blotting Minimal Reporting Standard (WBMRS)
Source: PLoS One. 2015 Aug 19;10(8):e0135392. doi: 10.1371/journal.pone.0135392 (PMC4545415; doi:10.1371/journal.pone.0135392)
Supplement: S1 Text — (DOCX) [file pone.0135392.s002.docx]

**S1 Text**

Sample Template for Western blot method incorporating the WBMRS.

Protein samples (___ µg/well) were separated on _____________ Gels (___ %) and transferred to _________ membranes (catalog #, manufacturer) using ____________ for ______ min. Western blotting procedures were carried out at _____°C. Membranes were blocked with __________ (catalog number, manufacturer) in _____ (reagent and composition) for ________ min. Membranes were then incubated with ____________ (primary antibody’s name, catalog number, manufacturer, lot number for polyclonal antibodies) in TBST with 1% non-fat milk at _______°C for ______ min or overnight. Total proteins on membranes were detected using ____________________ or protein normalization was carried out using anti-_______ at a dilution of 1:_______ (catalog #, manufacturer, lot number for polyclonal antibodies). Removal of excess primary antibody was carried out by washing the membranes in TBST three times for 5 min each. The secondary antibody anti-______ (catalog number, manufacturer) was diluted ______ and incubated with the membrane in ______ for _____ min at _______ temperature. Excess secondary antibody was removed by washing the membranes in ______ (buffer) ____ (number of times) times for ______ min each. Membranes were exposed to _______ ECL reagent (Cat. #, manufacturer for ______ min at _____ temperature and then visualized using a _______ MP (Cat. #, manufacturer). The target protein was semi-quantified using ____________ (version number and manufacturer). Background correction was carried out by ____________________________________ (if any).

Optional: Information about controls or validations done. For example, purified mouse ISG15 was used as a positive control while an ISG15 knock-out mouse model was used as the negative control. Validations could also include the use of immunizing peptides to determine if the antibody-antigen interaction is specific.
